# A Year's Work in the Hospitals and Medical Charities of London

**Published:** 1892-06-18

**Authors:** 


					June 18, 1892. THE HOSPITAL. 189
The Institutional Workshop.
A Year's Work in the Hospitals and Medical Charities of London
The following figures relate to the year 1891, and have been prepared in the office of the Metropolitan Hospital Sunday
Fund :?
NEWINGTON AND SOUTH DISTRICT,
Comprising Battersea, Wandsworth, Tooting, Balham, Streatham, Brixton, Lambeth, Ne wing ton, Southwark,
Bermondsey, Camberwell, Greenwich, Deptford, Lewiaham, Blackheath, Woolwich, &c.
No. of
Beds
Daily
Occu-
pied.
433
20
220
60
44
19
9
48
ilding
2
210
1,065
1,065
Hospitals.
la-
patients.
Oat-
patients.
Total
Expendi-
ture.
Income.
Chari-
table.
Pro-
prietary.
Patients'
Payments.
Total
Income.
Legacies
not
included
in
previouj
column.
Guys'
Miller
Seamen's
Evelina, for Children
Home for Sick Children
General Lying-in ...
Clapham Maternity...
Royal, for Children and Women
Royal South London Ophthalmic
Hospital for Diseases of the Skin
Metropolitan Convalescent
Dispensaries.
Battersea, Provident
Brixton, &c
Camberwell, Provident
Clapham ... ... '
Deptford Medical, &c.
Forest Hill
Gipsy Hill
Royal South London
South Lambeth, &c.
South London Medical Aid
Wandsworth Common
6,136
208
3,152
675
221
463
162
586
12
3,301
14,916
59,846
10,773
10,358
5,463
1,551
1,248
267
7,126
6,876
5,443
?
38,167
3,483
15,265
6,217
1,525
3,007
707
4,085
882
953
6,466
14,916
108,951
10,306
10,777
10,432
1,555
2,014
2,163
1,154
5,653
2,387
2,420
664
158,476
80,457
2,039
666
1,965
450
725
594
188
799
620
688
176
89,367
?
1,319
2,183
7,806
3,791
1,199
651
219
2,759
767
236
5,318
26,248
502
480
276
565
205
55
623
374
39
57
29 513
?
27,752
704
4,103
653
5
2,354
237
711
81
126
428
?
3,848
'"34
83
325
*208
190
"581
57
37,154
57
51
102
"94
111
6
158
37,733
5,326
1,886
105
1,390
129
17
370
133
"204
527
119
10,206
?
32,919
2,887
11,943
4,827
1,529
3,005
664
3,660
848
943
5,803
68,728
2,032
658
1,972
405
676
575
188
734
584
724
176
77,452
4,645
843
90
20
950
950
7,498
200
7,698
CITY AND EAST CENTRAL DISTRICT,
Comprising the City, St. Luke's, Shoreditch, Finabury, and Clerkenwell.
No. of
Beds.
No. of
Beds
Daily
Occu-
pied.
Hospitals.
78
160
80
55
36
34
100
27
16
586
69
130
44
40
22
21
80
18
12
436
Metropolitan
Royal Free ...
Royal, for Diseases of the Chest
North-Eastern, for Children
City of London Lying-in ...
St. Mark's, for Fistula
Royal London Ophthalmic
City Orthopaedic
Central London Throat and Ear
Dispensaries.
City
City of London and East London
Farringdon General
Finsbury ...
Metropolitan
Royal General
586
436
In-
patients.
880
1,866
499
508
455
311
1,892
150
228
Out-
patients.
Total
Expendi-
ture.
Income.
Total
Income.
Legacies
not
included
in
previous
column.
Ohari-
tible.
Pro- Patients'
prietary. Payments.
16,085
24,589
6,158
13,772
1,555
843
25,558
2,000
6,675
?
8,448
11,031
6,321
6,060
3,626
2,176
6,334
1,329
2,083
190
THE HOSPITAL
June 18, 1892.
ST. MARYLEBONE AND WEST CENTRAL DISTRICT,
Comprising St. Marylebone, St. John's Wood, Bloomsbury, Holborn, &c.
No. of
Beds.
60
20
51
307
81
30
179
16
58
42
53
170
25
13
18
35
94
25
1,273 | 1,909
No. of
Bed*
Daily
Occu-
pied.
51
12
46
254
81
29
130
8
37
24
47
156
19
4
2
34
64
11
Hospitals.
French
Italian
SS. John and Elizabeth
The Middlesex
Alexandra for Children
Home for Incurable Children
Hospital for Sick Children
British Lying-in
Queen Charlotte's Lying-in
New Hospital for Women...
Samaritan Free
National for the Paralysed, &c.
Hospital for Epilepsy, &c....
Central London Ophthalmic
W estern O phthalmic
National Orthopedic
London Homoeopathic
Establishment for Gentlewomen
National Dental
Dispensaries.
London Medical Mission ...
Margaret Street Infirmary
Marylebone Medical Mission
Portland Town
Portobello Road
St. John's Wood Provident
St. Marylebone General ...
Western General
In-
patients.
731
230
107
3,018
154
34
1,495
170
942
304
572
839
103
157
33
127
798
84
Out-
patients.
Total
Expetdi-
ture.
5,049
3,770
38,318
90
20,112
508
1,148
4,435
7,073
3,938
708
8,195
2,420
710
9,763
23,197
?
4,493
858
1,721
29,707
2,851
1.221
12,375
1,567
3,756
2,875
6,913
11,513
1,849
840
541
1,173
5,242
2,247
683
9,898
9,898
129,434 92,425
3.979
2.343
4,605
1,974
331
4,283
4 074
22,972
960
436
568
185
109
529
760
1,406
Income.
Chari-
table.
173,995 97,378
?
3,539
1,302
1,149
8,735
1,572
562
9,129
688
2,521
1 829
5,804
5,740
963
1,373
401
668
1,704
1,078
637
49,394
809
304
282
136
21
205
351
1,191
Pro-
prietary.
Patients'
Payments,
?
49
87
767
8,944
137
22
2,320
978
347
177
92
1,491
98
55
133
2,*425
108
18,230
14
149
100
3
4
26
133
39
52,693 18,698
Total
Income.
564
526
5
52
755
2,141
650
282
154
426
400
850
64
6,869
14
11
57
263
285
7,499
?
3,588
1,389
1,916
17,679
2,273
1,110
11,449
1,671
2,920
2,761
5,896
9,372
1,711
1,710
688
1,094
4,529
2,036
701
74,493
823
453
396
150
82
494
769
1,230
Legacies
not
included
in
previous
column.
184
9,912
600
4,335
600
310
2000
2,329
220
20
477
20,985
200
100
78,890 21,285
KENSINGTON AND WEST DISTRICT,
Comprising Kensington, Paddington, Bayswater, Kilburn, Chelsea, Brompton, Fulham, Hammersmith, Chiswick,
Brentford, Acton, Ealing, &c.
No. of
Beds.
356
281
101
321
23
50
27
102
60
105
135
20
30
12
1,623
No. of
Beds
Daily
Occu-
pied.
324
241
89
252
10
50
19
68
38
76
96
13
27
10
1,313
1,623 1 J,313
Hospitals.
St. George's
St. Mary's
West London
Hospital for Consumption
Belgrave, for Children
Cheyne, for Sick & Incurable Children
Paddington Green, for Children ...
Victoria, for Children
Chelsea, for Women
Cancer
Female Lock...
Male Lock
St. Mary Magdalene's Home
The Queen's Jubilee
Dispknsaries.
Brompton Provident
Chelsea, &c
Kensal Town, Provident
Kensington
Kilburn, Maida Vale
Kilburn Provident ...
Notting Hill Provident
Paddington Provident
Pimlico Provident ...
Royal Pimlico Provident
Westbourne Provident
In-
patients.
4,054
3,922
1,412
1,594
138
72
470
1,081
494
682
647
264
52
130
15,012
15.012
Out-
patients,
26,803
30,441
23,556
13,392
2,141
11,609
15.241
3,539
1,106
3,822
8,115
139,665
1,069
4.707
688
4,599
3,389
5,701
1,189
6,110
2,460
11.784
2,471
183 832
Total
Expendi-
ture.
?
35,755
22.905
6,674
27,819
1,330
2,299
2,332
6,550
4,378
9,929
5,005
1,761
848
1,771
128,756
480
644
322
709
475
1,048
215
579
487
901
388
135 004
Chari-
table.
?
10,304
10,028
4,974
14,495
1,150
2,169
1,958
5,197
4 029
4,488
2,743
228
336
1,158
63,257
157
484
39
553
533
169
126
155
18
363
69
65 923
Income.
Pro-
prietary.
?
15,158
2,765
106
8,767
32
134
235
489
65
1,575
77
8
29,411
56
174
47
45
53
75
"*22
15
43
29,941
Patients'
Payments
506
123
314
553
1,363
1,486
355
98
4,798
266
*224
894
90
370
438
552
301
7.933
Total
Income.
?
25,462
12,793
5 080
23 262
1,182
2,809
2,316
6,000
4,647
6,063
4,106
1,714
768
1,264
97,466
479
658
310
59S
586
1,138
216
547
456
930
413
103,797
Legacies
not
inclnded
previous
column.
?
27,229
5,256
14,332
210
5 000
360
297
600
9,655
925
100
63,994
63,994
June 18, 1892.
THE HOSPITAL.
191
ISLINGTON AND NORTH-WEST DISTRICT,
Comprising Islington, Holloway, Highbury, Hampstead, Highgate, St. Pancras, Stoke Newington, Tottenham, &c.
No. of
Beds
Daily
Occu-
pied.
66
45
59
182
47
57
62
11
21
550
550
Hospitals.
Great Northern Central ...
North West London
Tottenham
University College
North London Consumption
London Fever
London Temperance
Hampstead Home Hospital
Invalid Asylum
Dispensaries.
Camden Provident
Child's Hill
Hampstead Provident
Holloway and North Islington
Islington
St. Pancras and Northern...
Stamford Hill, &c. ...
In-
patients.
1,072
646
796
3,465
375
515
751
164
216
8,000
Out-
patients.
17,350
14,190
6,397
44,831
2,810
5,462
91,040
853
915
10,496
3,977
12,232
2,190
4,742
Total
Expendi-
ture.
?
6,184
3.791
4,598
20,396
5,402
8,420
8,171
2,334
944
60,240
322
251
900
851
969
653
614
8,000 I 126,445 I 64,800 II 37,196
Income.
Chari-
table.
?
3,348
4,871
3,101
6.251
4,341
6,493
5,977
325
553
35,260
41
42
320
407
277
367
482
Pro-
prietary.
?
414
123
879
4,700
85
2,071
885
745
121
10,023
10
16
44
6
103
120
10,322
Patients'
Payments,
105
532
84
15
2,062
782
605
221
3,806
276
199
' 572
339
551
92
5,835
Total
Income.
?
3,762
5,099
4,512
11,035
4,441
10,626
7,044
1,675
895
49,089
317
251
908
790
834
562
602
53,353
Legacies
not
included
in
previous
column.
?
1.319
381
7.320
120
2,483
680
1,131
13,434
542
1,036
15,012
WESTMINSTER DISTRICT,
Comprising Westminster City and Liberties.
140
186
164
108
13
51
23
25
50
18
5
15
826
826
ChariDg Cross
King's College
Westminster
Ventnor, for Consumption
Grosvenor, for Women & Children
Hospical for Women
National, for Diseases of Heart, &c.
Royal Westminster Ophthalmic ...
Royal Orthopaedic
Hospital for Diseases of the Throat
Royal Ear I
Dental
Gordon, for Fistula
St. Peter's, for Stone
Dispensaries.
Public
St. George and St. James ...
St. George, Hanover Square
Western
W estmins ter General
2,291
2,075
2,557
722
108
670
126
405
154
526
153
123
367
10,277
21,243
21,461
26,607
1,715
5,375
1,796
8,985
1,032
7,260
2,742
28,105
498
7,750
134,569
3,284
3,465
1,482
3,210
4 679
?
15,040
19,145
14,230
10,941
1,554
6,701
2,077
2,236
2,130
4,810
754
1,919
873
3,461
85,871
736
523
619
1,152
490
?
7,213
9/206
4,567
4,318
804
2,948
1,661
1,729
1,276
878
114
2,193
453
681
10,277 ! 150,689 I 89 391 || 40,282 ' 11,461 11,320
38,041
764
382
417
374
304
?
3,025
1,385
3,015
1,483
12
191
370
408
168
4
184
492
73
2,999
404
949
427
40
237
2,406
566
382
372
1,852
10,737
160
17
20
381
146
10,707
38
145
373
57
?
10,238
10,664
7,382
9,000
1,220
4,088
2,088
2,139
1,921
3,452
684
2,759
825
3,025
59,485
924
437
582
1,128
507
?
3,419
4,908
3,120
1,104
8,052
686
516
100
900
*50
22,855
*504
63 063 1 23 359
STRATFORD AND EAST-END DISTRICT,
Comprising Bethnal Green, Tower Hamlets, West Ham, Whitechapel, Haokney,
and the East.
Stepney, Limehouse, Poplar,
125
776
42
164
102
92
12
1,313
96
612
31
122
84
68
4
1,017
1,313 1.017
German
London
Poplar
City of London for DIs. of the Chest
East London for Children ...
Mrs. Gladstone's Home ...
East End Mothers' Home ...
Dispensaries.
Eastern
Hackney Provident.
London
Queen Adelaide's
Tower Hamlets
West Ham, &c.
1,301
8,842
570
1,102
1,107
1,047
86
14,055
253
14.308
22,784
126,587
12,312
14,579
21,216
28
197,506
7,200
636
2,289
4,878
4,498
24,699
?
9,541
74,227
3,364
10,441
7,695
1,441
897
107,606
756
234
460
497
528
2,668
241.706 112 649
?
7,121
18,734
3,246
6,016
7.963
772
648
44,500
343
7
144
561
376
2,814
48.745
?
2,404
23,852
397
472
434
368
9
27,936
314
266
180
43
160
28 899
?
329
440
776
71
252
"37
116
?
9,854
43 026
3,643
6,488
8,397
1,140
664
?
316
6,678
196
1,106
1,339
73,212 I 9,635
728
259
410
778
535
2,974
325
1,252 78 896 I 9.960
192
THE HOSPITAL.
June 18, 1892.
SUMMARY.
It will be seen from the following summary that the Voluntary Hospitals and Medical Charities of London, during the
12 months ending 31st December, 1891, relieved over one million two hundred and sixty thousand patients at a cost of
?641,533. The Ordinary Income only amounted to ?494,358, leaving a deficiency of ?147,175 on the year's work.
No. of
Beds.
1,592
586
993
1,273
1,623
835
1,313
8,215
No. of
Beds
Daily
Occu-
pied.
1,065
436
826
1,009
1,313
550
1,017
6.216
Hospitals and Dispensaries.
Newington and South District ...
City and East Central District ...
Westminster District
St. Marylebone and West Central
District
Kensington and West District ...
Islington and North-West District
Stratford and East-End District...
In-
patients.
14,916
6,789
10.277
9,898
15,012
8,000
14,308
79,200
Out-
patients.
158,476
145,702
150,689
173,995
183,832
126,445
241,706
1,180,845
Total
Expendi-
ture.
?
89,367
52,944
89,391
97,378
135,004
64,800
112,649
641,533
Chari-
table.
?
29,513
26,558
40,282
52,693
65,923
37,196
48,745
300,910
Income.
Pro-
prietary.
Patients*
Payments.
?
37,733
7,574
11,461
18,698
29,941
10,322
28,899
144,628
?
10,206
4,775
11,320
7,499
7,933
5,835
1,252
48,820
Total
Income:
?
77,452
38,907
63,063
78,890
103,797
53,353
78,896
494,358
Legacies
not
included
in
previous
column.
?
7,698
8,025
23,359
21,285
63,994
15,012
9,960
149,333

				

